# Supplementary material for: Perturbation-based gene regulatory network inference to unravel oncogenic mechanisms
Source: Sci Rep. 2020 Aug 25;10:14149. doi: 10.1038/s41598-020-70941-y (PMC7447758; doi:10.1038/s41598-020-70941-y)
Supplement: Supplementary file 1 — Supplementary Information. [file 41598_2020_70941_MOESM1_ESM.pdf]

Supporting Information for 'Perturbation-based gene regulatory network inference to unravel oncogenic mechanisms'

Daniel Morgan[1], Matthew Studham[1], Andreas Tjärnberg[1,2], Holger Weishaupt[3], Fredrik J. Swartling[3], Torbjörn E. M. Nordling[4]*, Erik L.L. Sonnhammer[1]*

[1]Department of Biochemistry and Biophysics, Stockholm University, Science for Life Laboratory, Box 1031, 17121 Solna, Sweden. [2]Lionel Lab, Center for Developmental Genetics, New York University. [3]Rudbeck Laboratory, Department of Immunology, Genetics and Pathology, Science for Life Laboratory, Uppsala University, Uppsala, Sweden. [4]Department of Mechanical Engineering, National Cheng Kung University, Taiwan.

* Corresponding authors Erik.Sonnhammer@SciLifeLab.se, torbjörn.nordling@nordlinglab.org

Perturbation-based gene regulatory network inference to unravel oncogenic mechanisms

Daniel Morgan[1], Matthew Studham[1], Andreas Tjärnberg[1,2], Holger Weishaupt[3], Fredrik J. Swartling[3], Torbjörn E. M. Nordling[4]*, Erik L.L. Sonnhammer[1]*

[1]Department of Biochemistry and Biophysics, Stockholm University, Science for Life Laboratory, Box 1031, 17121 Solna, Sweden. [2]Lionel Lab, Center for Developmental Genetics, New York University. [3]Rudbeck Laboratory, Department of Immunology, Genetics and Pathology, Science for Life Laboratory, Uppsala University, Uppsala, Sweden. [4]Department of Mechanical Engineering, National Cheng Kung University, Taiwan.

* Corresponding authors Erik.Sonnhammer@SciLifeLab.se, torbjörn.nordling@nordlinglab.org

Supporting Information Legends

S1 Fig. Comparison of the two technical replicates in terms of qPCR cycles.

S2 Fig. Volcano plot describing fold change and significance of the knockdown responses.

S3 Fig. Independent validation of inferred GRNs' topologies. Each x-axis tick mark shows the prediction performance in terms of the wRSS error of each inferred GRN topology (circles) fit to independent validation data under cross-validation, compared to its shuffled topologies. The box displays the median and interquartile range, and whiskers bound points maximally extending 1.5 times this range. Beyond this, outlier points are shown.

S4 Fig. Independent validation of inferred GRNs' fit to measured data. Each x-axis tick mark shows the prediction performance in terms of the wRSS of an inferred GRN topology fit to independent validation data under cross-validation, compared to its ability to fit shuffled data. X marks represent the inferred GRNs. The filled color box displays the median and interquartile range, and whiskers bound points maximally extending 1.5 times this range. Beyond this, outlier points are shown.

S5 Fig. Predictiveness of inferred GRN topologies using R^2 for the same GRNs as in Fig 2.

S6 Fig. Experimental validation of the predicted activation of CCNB1 by BRD4. CCNB1 expression was measured as $\log_{10}$ of counts per million reads after 2 and 6 hours of exposure to control (DMSO) and JQ1, which inhibits BRD4. The effect on CCNB1 was significant at both time points (p=0.048 and 0.014 by t-test).

S7 Fig. Experimental validation in the human adult high-grade glioma U3056 cell line of the predicted activation of CCNB1 by BRD4. CCNB1 expression was measured as $\log_{10}$ of counts per million reads after 6 hours exposure to control (DMSO) and JQ1, which inhibits BRD4. The effect on CCNB1 was significant (p=0.005 by t-test).

Supporting Information

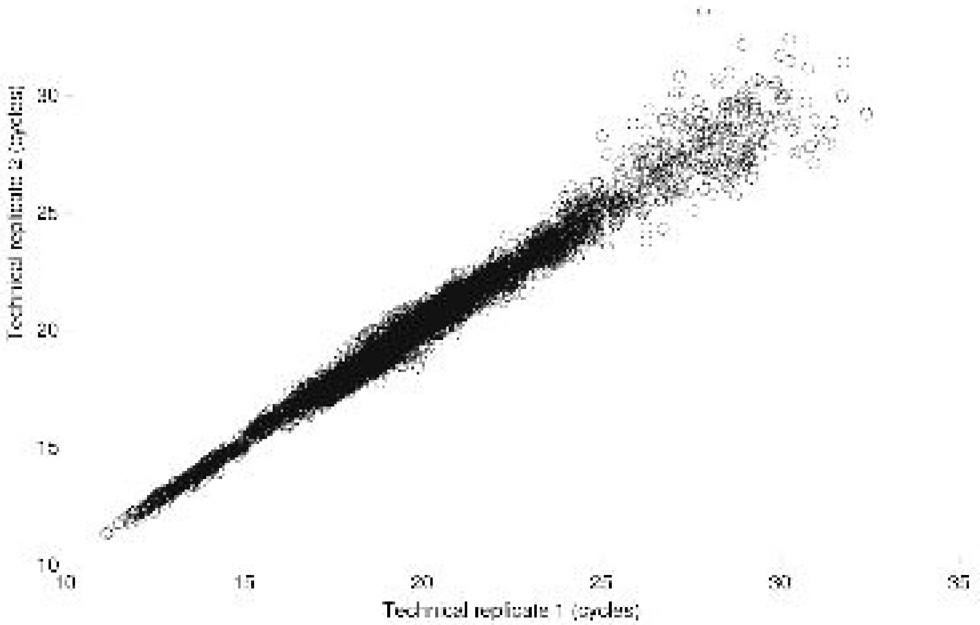

S1 Fig. Comparison of the two technical replicates in terms of qPCR cycles.

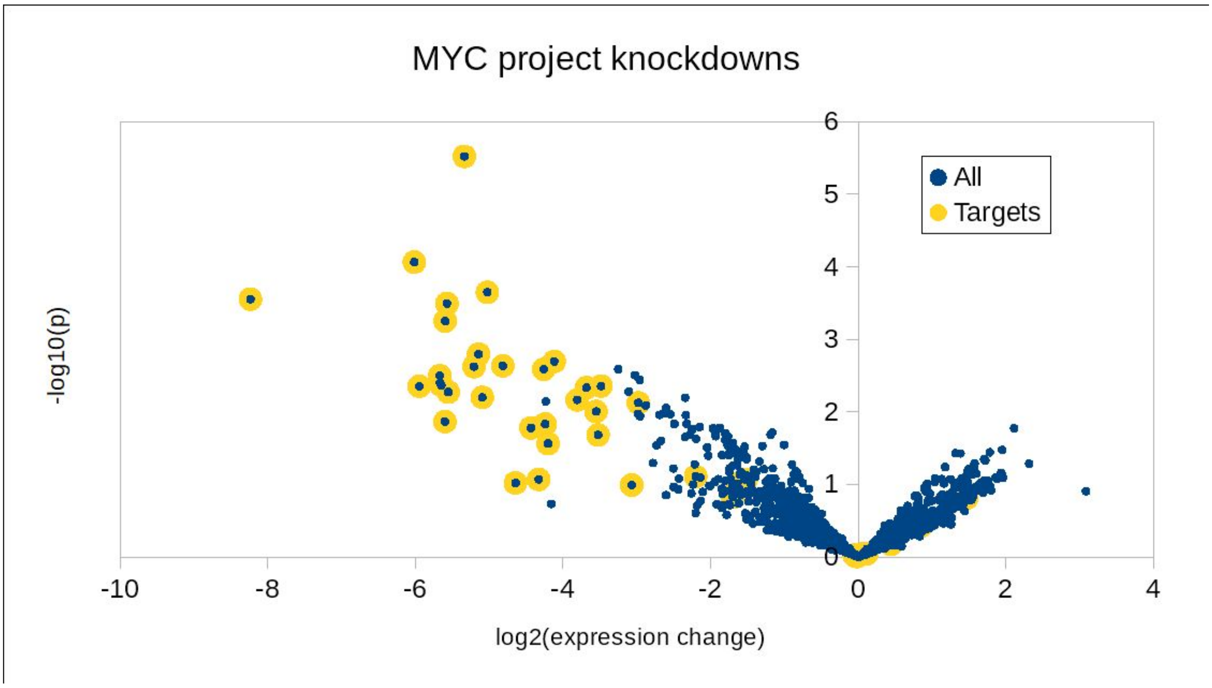

S2 Fig. Volcano plot describing fold change and significance of the knockdown responses.

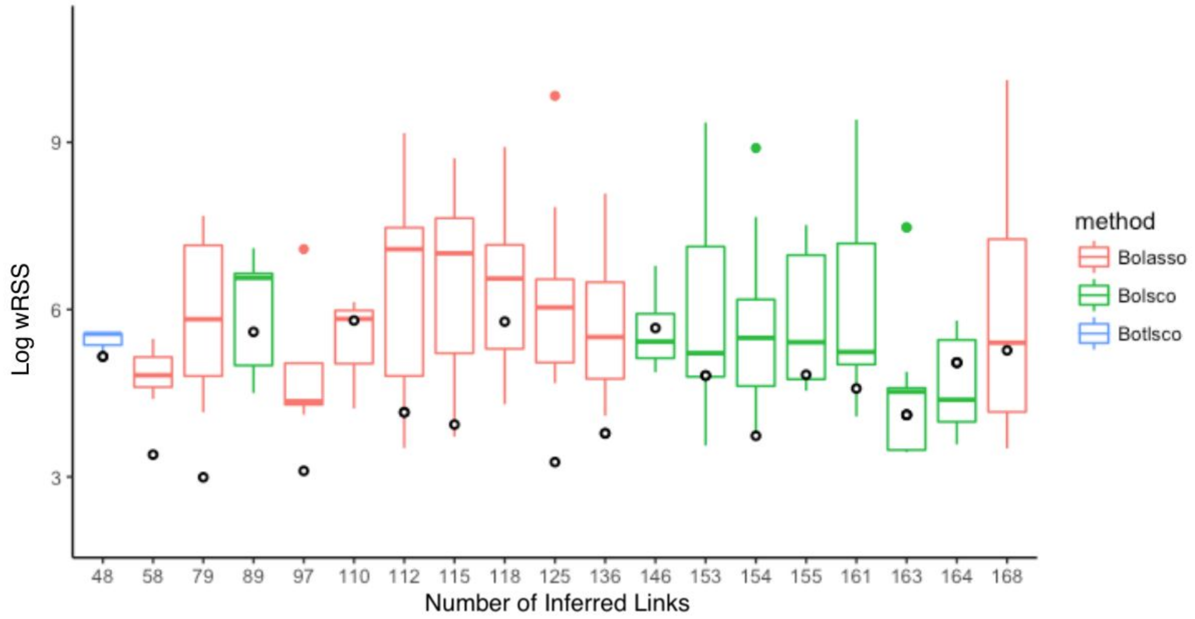

S3 Fig. Independent validation of inferred GRNs' topologies. Each x-axis tick mark shows the prediction performance in terms of the wRSS error of each inferred GRN topology (circles) fit to independent validation data under cross-validation, compared to its shuffled topologies. The box displays the median and interquartile range, and whiskers bound points maximally extending 1.5 times this range. Beyond this, outlier points are shown.

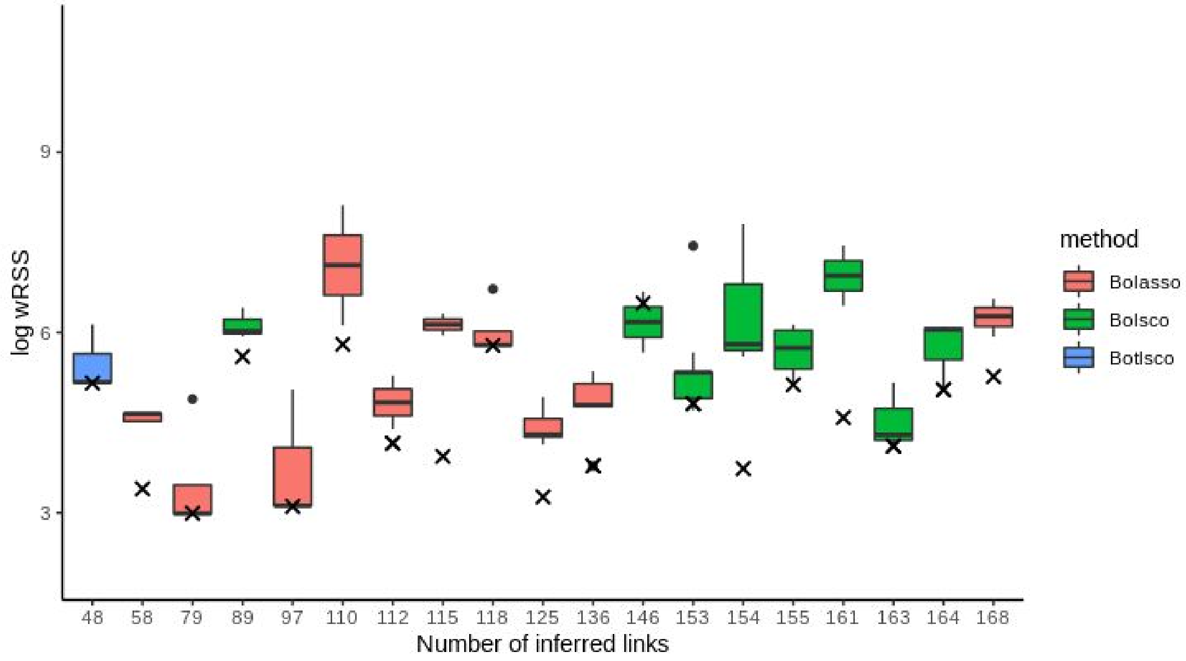

S4 Fig. Independent validation of inferred GRNs' fit to measured data. Each x-axis tick mark shows the prediction performance in terms of the wRSS of an inferred GRN topology fit to independent validation data under cross-validation, compared to its ability to fit shuffled data. X marks represent the inferred GRNs. The filled color box displays the median and interquartile range, and whiskers bound points maximally extending 1.5 times this range. Beyond this, outlier points are shown.

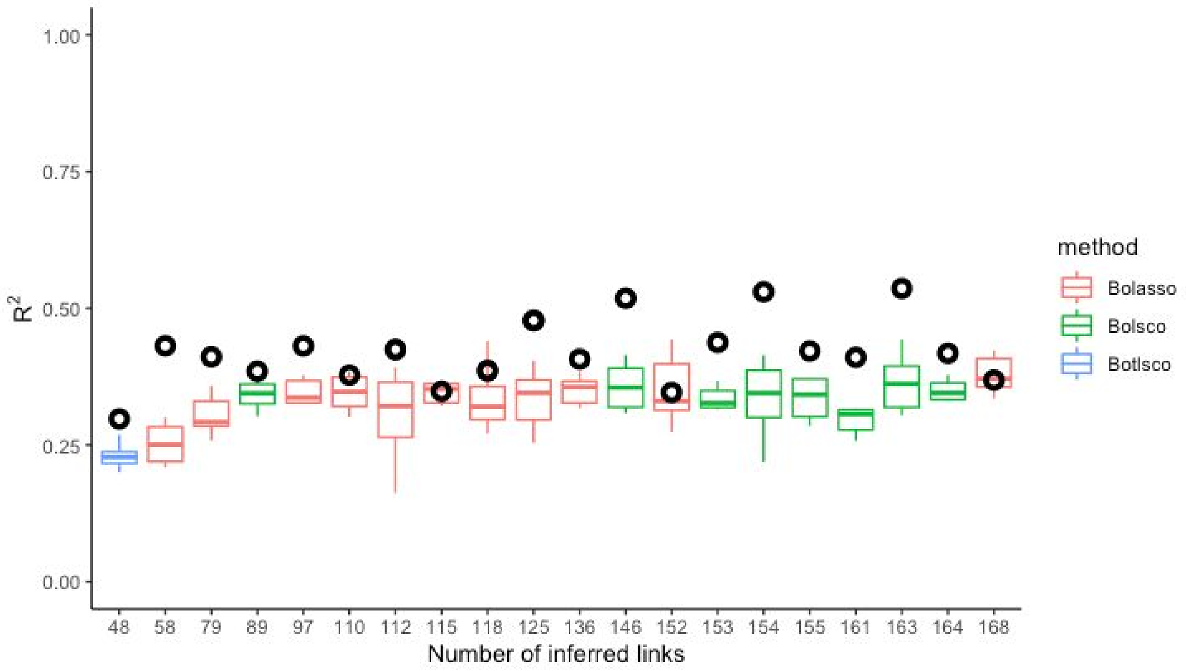

S5 Fig. Predictiveness of inferred GRN topologies using R^2 for the same GRNs as in Fig 2.

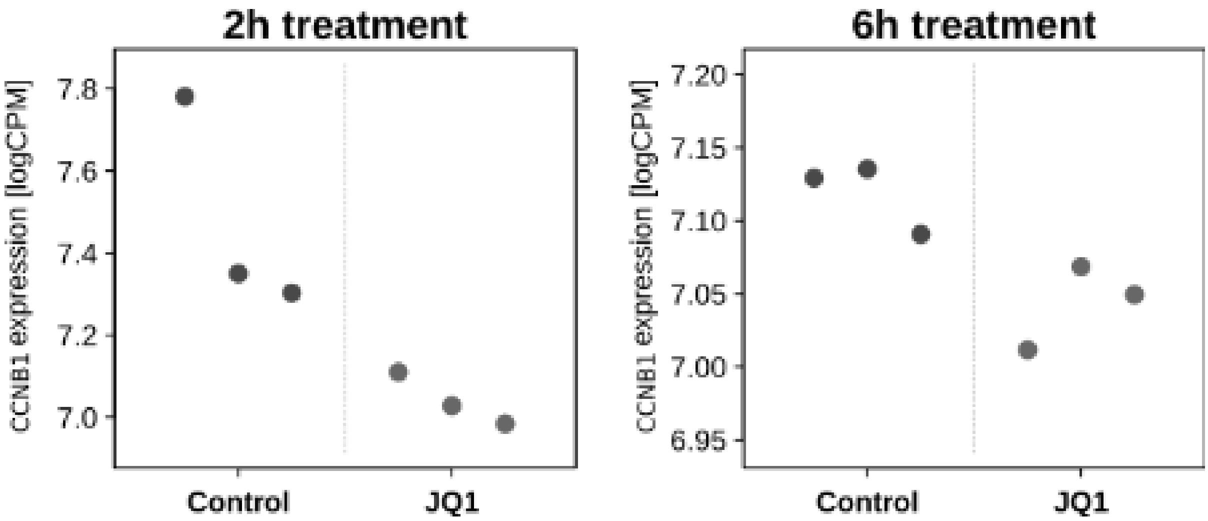

S6 Fig. Experimental validation in the GTML2 cell line of the predicted activation of CCNB1 by BRD4. CCNB1 expression was measured as log_{10} of counts per million reads after 2 and 6 hours of exposure to control (DMSO) and JQ1, which inhibits BRD4. The effect on CCNB1 was significant at both time points (p=0.048 and 0.014 by t-test).

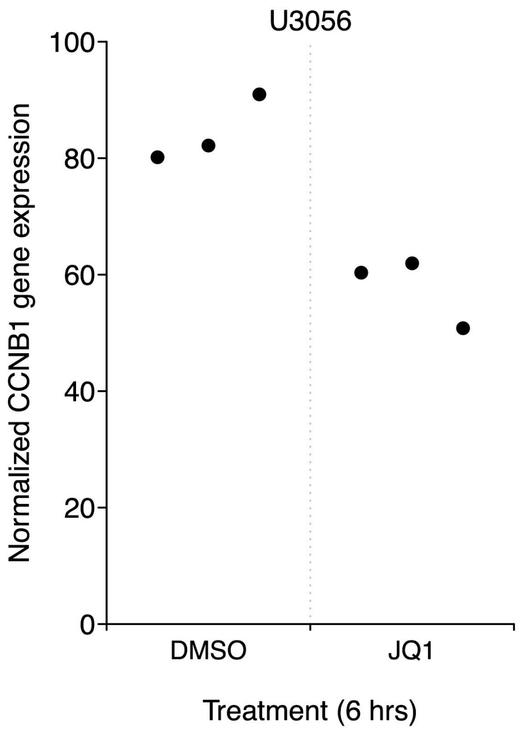

S7 Fig. Experimental validation in the human adult high-grade glioma U3056 cell line of the predicted activation of CCNB1 by BRD4. CCNB1 expression was measured as $\log_{10}$ of counts per million reads after 6 hours exposure to control (DMSO) and JQ1, which inhibits BRD4. The effect on CCNB1 was significant (p=0.005 by t-test).

S1 Table. Description of matrix contents used in the linear model for GRN inference.

**matrix**	**dimension (rows x columns)**	**contents**	**description/ value range**
Y	40 x 115	Gene expression	SNR=0.014, max=3.3, min=-8.7, condition number=55.3
A	40 x 40	Interaction matrix (GRN)	Range dependent on BFECV per init; initial inference returns variously sparse, weighted A, which is reweighted (see BFECV section)
P	40 x 115	Perturbation/ design	Targeted perturbations (-1=KD, 0=no KD)
F	40 x 115	Process error	Range dependent on BFECV per init
E	40 x 115	Measurement error	Range dependent on BFECV per init

S2 Table. Genes investigated. Description of perturbed genes in terms of pathways involved in, complexes formed, general notes (such as affect on Myc transcription), and RPKM expression in cell line A431. Pathway source material is the NCBI BioSystems Database unless otherwise noted. Complex information and General Notes are taken from NCBI. Gene names are listed as in our results and then synonyms in HGNC.

Genes & synonyms	Pathway	Complex	Functional Annotation	RPKM
**AES**	ps1pathway	CtBP/CBP/TCF1/TLE1/AES, beta catenin/TCF1/CtBP/CBP/TLE1/AES/SMAD4	Transcriptional corepressor/ regulation and signaling. First complex represses MYC, second activates MYC	51.5
**AIB1 / NCOA3**	Era genomic pathway	E2/ERA (dimer)/AIB1	Transcriptional regulation and enzymatic behavior. Nuclear receptor coactivator. Complex activates MYC	15.1
**AKT1**	Il2 pi3kpathway	AKT1+plasma membrane	Kinase, enzyme, oncogene, metabolism. ser/thr kinase that activates MYC when in plasma membrane, oncogene	13.2
**BIRC5**			Transcription regulation. Protease inhibitor, Repressor	44.1
**BRCA1**	Myc repression pathway		Transcription regulation,DNA-binding, metabolic and enzymatic activity, Tumor suppressor. Works w/MYC to repress psoriasin, tumor suppressor gene	16.8
**BRD4 / (M)CAP / HUNK(L)**	PI3K/ AKT pathway	activates Myc	Transcription regulation, metabolic and enzymatic activity, common complex w/P-TEFb (Delmore 2011[26]), oncogene	8.1
**BYSL**			[45,83]	15.5
**CCNB1**	FoxO signaling pathway		metabolic activity, Cyclin B1 Ser/thr kinase activator	145
**CEBPB**	MEK/ERK signaling pathway		Transcription factor, DNA-binding	5.3
**CHUK**			Serine kinase, metabolic	14.6

			activity,	
**CTNNB1**	BDNF signaling pathway<br>Wnt signaling pathway		Transcription regulation, signaling, enzymatic activity,oncogene	100.5
**DP1**	e2f pathway	E2F1-3/DP	Transcription regulation, DNA-binding, complex activates MYC, oncogene, tumor suppressor gene	6.6
**DP2**	e2f pathway	E2F4/DP2/TRRAP, E2F4/DP2/GCN5	Transcription regulation, DNA-binding, complexes activate MYC	15.2
**DVL3**	Beta catenin nuc pathway	TCF4/beta catenin/JUN/DVL3	Signaling, complex activates Myc	18.3
**E2F4**	E2f pathway	E2F4/DP2/TRRAP, E2F4/DP2/GCN5, CEBPA/BRM/RB1/E2F4	Transcription regulation, DNA-binding, First two complexes activate MYC, the second represses MYC	15.2
**ENO1**	Myc active pathway<br>Notch pathway		Transcription regulation, DNA-binding, metabolic activity, represses MYC	919.1
**FOXO3A / FOXM1C**	Myc represions pathway		Transcription regulation, DNA-binding, enzymatic activity, Tumor suppressor. [58] [58]	13.3
**GCN1(L1)**			[73]	58.3
**GNL3**			[47] [47]	46.3
**JUN***	Ap1 pathway	JUN/JUND	activates as JUN/JUND	15.3
	Betacatenin nuc pathway	TCF4/beta catenin/JUN/DVL3	Transcription regulation, DNA-binding, enzymatic activity, complex activates MYC, oncogene	
**LKB1 / STK11**	Lkb1 pathway	LKB1/ER alpha	complex activates MYC, Kinase, metabolic activity, Tumor suppressor.	10
**MAX**	Myc activation pathway	MYC/Max/RPL11,MYC/Max/TRRAP/TIP60/TIP49A/TIP49B/BAF53		21.7
	Myc pathway	replication preinitiation complex, MYC/Max/TRRAP/TIP60/	Transcription regulation, DNA-binding, Tumor suppressor.	

		TIP49A/TIP49B/BAF53		
	Myc repression pathway	SMAD2-3/SMAD4/MYC/Max/MIZ-1,MYC/Max/NF-Y,MYC/Max/MIZ-1/ZO2, MYC/Max		
**MIZ(-)1 / ZBTB17**	Myc pathway	MYC/Max/MIZ-1	Transcription regulation, DNA-binding, Main MYC complex that represses	2.5
	Myc repression pathway	MIZ-1/p300,MYC/Max/MIZ-1,MYC/Max/MIZ-1/DNMT3A/GFI1		
**MYB**	Cmyb pathway	Myb, SKI/SIN3a/NCoR1/TIF1B/Myb	Transcription regulation, DNA-binding, metabolic and enzymatic activity, enzymatic activity, activates as Myb; represses MYC as part of complex, oncogene	1.5
**MYC**	Myc activation pathway	MYC/Max/RPL11,MYC/Max/TRRAP/TIP60/TIP49A/TIP49B/BAF53		20.7
	Myc pathway	MYC/Max/TRRAP/TIP60/TIP49A/TIP49B/BAF53, MYC/Max	Transcription regulation, DNA-binding, oncogene,	
	Myc repression pathway	MYC/Max/NF-Y,MYC/Max/MIZ-1/ZO2,MYC/Max		
**MYCBP**	Notch pathway	MYCBP	Transcription regulation, binds to MYC, stimulates E-box transcription activation	30.7
**NFKB1**	Cd40 pathway	RelA/NFkappaB1	Transcription regulation, DNA-binding, complex activates MYC	15.9
**P107 / PRB1 / RBL1**	Myc repression pathway		works w/MYC/Max to repress MYC	15.1
**(E)P300**	Myc repression pathway	MIZ-1/p300	Transcription regulation, metabolic activity, Tumor suppressor. Makes complex w/MIZ	13.6
**(T)P53**	Myc activation pathway		Transcription regulation, DNA-binding, enzymatic activity, oncogene, tumor suppressor.	26.9
**RB1**	E2f pathway	CEBPA/BRM/RB1/E2F4	Transcription regulation, DNA-binding, Tumor suppressor. complex represses MYC	29.3
**RELA**	Cd40 pathway	RelA/NFkappaB1	Transcription regulation, DNA-binding, complex	19.1

			activates MYC	
**RPL11**	Myc activation pathway	MYC/Max/RPL11		716.1
**SP1**	Foxm1 pathway	FOXM1C/SP1	Transcription regulation, DNA-binding, complex works against MYC,tumor suppressor gene	21.4
	Myc repression pathway	SMAD2-3/SMAD4/SP1		
**STAT3**	Il6 7pathway	STAT3 dimer	Transcription regulation, DNA-binding, enzymatic activity, dimer activates MYC, oncogene	19.9
**STAT5 A/B**	Il2 stat5pathway	STAT5 dimer	Transcription regulation, DNA-binding, metabolic activity, dimer activates MYC (ENCODE[30]), oncogene, tumor suppressor gene	7.3
**TBP**	Myc repression pathway		Transcription regulation, DNA-binding, works against MYC in affecting multiple genes	5.8
**TCF7L2**	Wnt signaling pathway, beta catenin nuc pathway	TCF4/beta catenin/TNIK, TCF4/beta catenin/JUN/DVL3	Transcription regulation, DNA-binding, enzymatic activity, signaling, complexes activate Myc (Sur 20[31]), oncogene	5.4
**TIF1B / TRIM28**	Cmyb pathway	SKI/SIN3a/NCoR1/TIF1B /Myb	Transcription regulation, metabolic activity, part of complex that represses	91
**YY1**	Notch pathway	NICD/YY1	Transcription regulation, DNA-binding, complex activates MYC	27.9

S3 Table. Comparing the best GRN to reference networks. The networks include an expected prior network gathered from data mining, as well as the TRRUST v2, FunCoup v4, HumanNet v1 and STRING v10.5 databases. P-values are from a hypergeometric test. *links with confidence >= 0.90

Network	Overlapping links	Links in DB	Links in GRN	p-value
Prior network	5	40	86	0.08
TRRUST	7	132	86	0.66
FunCoup	14	146	76	0.01*
HumanNet	9	81	76	0.02

STRING	21	212	76	0.003

S4 Table. Used siRNA sequences

Symbol	Entrez Gene	Ensembl Gene	Ambion siRNA ID	siRNA Sequences
AES	166	ENSG00000104964	ASX053MJ	GCCUCAAGCUCGAAUGUGATT CAAAGACGAAUUUCAGCUATT GAACAUCGAGAUGCACAAATT
AIB1	8202	ENSG00000124151	ASX04YYH	CAGUAUAUCGAUUCUCGUUTT CAGUAAGACAGAUACGUCATT GGGCUUUUAUUGCGACCAATT
AKT1	207	ENSG00000142208	ASX04WP6	GCGUGACCAUGAACGAGUUTT GAACAAUCCGAUUCACGUATT CGGUAGCACUUGACCUUUUTT
BIRC5	332	ENSG00000089685	ASX04ZH8	GGACCACCGCAUCUCUACATT GCAGGUUCCUUAUCUGUCATT CAAAGGAAACCAACAAUAATT
BRCA1	672	ENSG00000012048	ASX053MG	GACCCAGUCUAUUAAAGAATT CAGCUACCCUUCCAUCAUATT CAUGCAACAUAACCUGAUATT
BRD4	23476	ENSG00000141867	ASX051I6	UGAGCACAAUCAAGUCUAATT AGAUUGAAAUCGACUUUGATT CCUGAUUACUAUAAGAUCATT
BYSL	705	ENSG00000112578	ASX055LN	AGGUGGUUGUGGACCCUGATT CCAGGAUUUUUGCCUCUAATT GGGAGGUAUUAUCUAAGUATT
CCNB1	891	ENSG00000134057	ASX04ZMX	CAACAUUACCUGUCAUAUATT GAAAUGUACCCUCCAGAAATT CACUUAUACUAAGCACCAATT
CEBPB	1051	ENSG00000172216	ASX04ZP9	CCGCCUGCCUUUAAAUCCATT GGCCCUGAGUAAUCGCUUATT GUAUAUUUUGGGAAUCUUUTT
CHUK	1147	ENSG00000213341	ASX04WNH	GGACUAAAAGAAGACUAUATT GAAGGAUCCAAAGUGUAUATT GCCUAGAGCUAAGUACCAATT
CTNNB1	1499	ENSG00000168036	ASX04X6P	GGACCUAUACUUACGAAAATT GGAUGUUCACAACCGAAUUTT CUGUUGGAUUGAUUCGAAATT
DP1	7027	ENSG00000198176	ASX04XZ1	CAUCUCCAAUGACAAAUUUTT GCAAGAAGACGGUCAUCGATT GCUCCAAUGGGUCUCAGUATT
DP2	7029	ENSG00000114126	ASX04XZ2	GGUUAUGCUUGGAUGCAGATT GAUCCAUGAUGACAUAGAATT GGACUACUUCUGAACUCUATT
DVL3	1857	ENSG00000161202	ASX04X6U	GGUAAACGAGAUCAACUUUTT CCAGCUUCUUUGACUCAGATT CGGUCACUCUCAACAUGGATT
E2F4	1874	ENSG00000205250	ASX04ZWZ	AGAAAUCUUUGAUCCCACATT GUAUCGGGCUAAUCGAGAATT GGAUUUACGACAUUACCAATT
ENO1	2023	ENSG00000074800	ASX04ZYQ	CCGUGACCGAGUCUCUUCATT GCAGGUACAUCUCGCCUGATT CAGUGGUGUCUAUCGAAGATT
FOXM1C	2305	ENSG00000111206	ASX0501W	GCUCAUACCUGGUACCUAUTT CACUAUCAACAAUAGCCUATT GGAUCAAGAUUAUUAACCATT
GCN1L1	10985	ENSG00000089154	ASX0518S	GGUGUAACCGAUUCACUAUTT GCAGCCUUGUUGCUCUUAATT GGCAGUUGAUUGUGGAUGATT
GNL3	26354	ENSG00000163938	ASX051NW	GGGUUGGAGUAAUUGGUUUTT GUAUUGUGGUAGACAUGAATT CCUCCGAUGUUGUCCUAGATT
JUN	3725	ENSG00000177606	ASX050EK	GGCACAGCUUAAACAGAAATT GGAUCAAGGCGGAGAGGAATT CCAAGUGCCGAAAAAGGAATT
LKB1	6794	ENSG00000118046	ASX04WWS	GGCUCUUACGGCAAGGUGATT ACAUCACCACGGGUCUGUATT AGGAGGUUACGGCACAAAATT
MAX	4149	ENSG00000125952	ASX050J3	CAAACGGGCUCAUCAUAAUTT CACACACACCAGCAAGAUATT CAAAGACAGCUUUCACAGUTT
MYB	4602	ENSG00000118513	ASX050LY	CCUCUCAUCUAGUAGAAGATT GGAAAAGCGAAUAAAGGAATT GAAUUGCUCCUAAUGUCAATT
MYC	4609	ENSG00000136997	ASX050M5	ACAGCCCACUGGUCCUCAATT GAGCUAAAACGGAGCUUUUTT AGACCUUCAUCAAAAACAUTT
MYCBP	26292	ENSG00000214114	s25391	GUAUGAAGCUAUUGUAGAATT UUCUACAAUAGCUUCAUACTT
NFKB1	4790	ENSG00000109320	ASX053S7	GGCUCAUGUUUACAGCUUUTT CCACCUUCAUUCUCAACUUTT GCAGCUGUAUAAGUUACUATT
P107	5933	ENSG00000080839	ASX04XXC	GCUCUUUGCCUAUAGCUCATT GGAUGGACAUGUCAAAUCUTT CCACCAAAGUUACCACGAATT
P300	2033	ENSG00000100393	ASX04ZYU	GGACUACCCUAUCAAGUAATT CCACUACUGGAAUUCGGAATT GCCUGGUUAUAUAACCGGATT
P53	7157	ENSG00000141510	ASX053MH	GUAAUCUACUGGGACGGAATT GAAAUUUGCGUGUGGAGUATT GGUGAACCUUAGUACCUAATT
RB1	5925	ENSG00000139687	ASX04XXA	GGAUAGCAAAACAACUAGATT CCAGUACCAAAGUUGAUAATT GCGUGUAAAUUCUACUGCATT
RELA	5970	ENSG00000173039	ASX04XXT	CCCUUUACGUCAUCCCUGATT GGAGUACCCUGAGGCUAUATT GGAUUGAGGAGAAACGUAATT
RPL11	6135	ENSG00000142676	ASX0571N	GGUGCGGGAGUAUGAGUUATT CAACUUCUCAGAUACUGGATT GGAACUUCGCAUCCGCAAATT
SP1	6667	ENSG00000185591	ASX04Y7O	GGCAGACCUUUACAACUCATT CCACAAGCCCAAACAAUCATT GCAACAUGGGAAUUAUGAATT

STAT3	6774	ENSG00000168610	ASX04Y94	GCACCUUCCUGCUAAGAUUTT GGCUGGACAAUAUCAUUGATT GCCUCAAGAUUGACCUAGATT
STAT5B	6777	ENSG00000173757	ASX04Y97	CACCCGCAAUGAUUACAGUTT GGAGACUUGAAUUACCUUATT GCAUCACCAUUGCUUGGAATT
TBP	6908	ENSG00000112592	ASX04XTS	CCAACAAUUUAGUAGUUAUTT GCCUAUUCAGAACACCAAUTT CAGUGAAUCUUGGUUGUAATT
TCF7L2	6934	ENSG00000148737	ASX04XU9	GAUGGAAGCUUACUAGAUUTT CAAUGAAUCAGAAACGAAUTT GGUCAACCAGUGUACCCAATT
TIF1B	10155	ENSG00000130726	ASX05101	GGCCCUAUUCUGUCACGAATT GCAACAUUGCAGAAGAGCATT GCGGAAAUGUGAGCGUGUATT
YY1	7528	ENSG00000100811	ASX053W2	AGCUUUUGUUGAGAGUUCATT AGAACUCACCUCCUGAUUATT CAGAAUAUAUGACAGGAAATT

S5 Table. TaqMan assays used for qPCR in Fluidigm Biomark

TATAA Assay	Gene	TaqMan Assay
AES	AES	Hs01081011_g1
AKT1	AKT1	Hs00920503_m1
BCL2	BCL2	Hs00236808_s1
BIRC5	BIRC5	Hs04194392_s1
BRCA1	BRCA1	Hs01556193_m1
BRD4	BRD4	Hs04188087_m1
BYSL	BYSL	Hs00608266_m1
CCNB1	CCNB1	Hs01030103_m1
CEBPB	CEBPB	Hs00270923_s1
CHUK	CHUK	Hs00989502_m1
CTBP1	CTBP	Hs00972284_m1
CTNNB1	CTNNB1	Hs00170025_m1
DVL3	DVL3	Hs00610263_m1
E2F4	E2F4	Hs00608098_m1
ENO1	ENO1	Hs00361415_m1
EP300	P300	Hs00914223_m1
FOXM1	FOXM1C	Hs01073586_m1
GCN1L1	GCN1L1	Hs00412445_m1
GNL3	GNL3	Hs00205071_m1
JUN	JUN	Hs01103582_s1
JUND	JUND	Hs00534289_s1
MAX_g1	MAX	Hs00811069_g1
MAX_m1	MAX	Hs01105523_m1
MYB	MYB	Hs00920556_m1
MYC	MYC	Hs00153408_m1
MYCBP-GJA+	MYCBP	Hs00429315_g1
MYCL	LMYC	Hs00607136_g1
MYCL1	LMYC	Hs00420495_m1
MYCN	NMYC	Hs00232074_m1

NCOA3	AIB1	Hs00180722_m1
NFKB1	NFKB1	Hs00765730_m1
RB1	RB1	Hs01078066_m1
RBL1	P107	Hs00765707_m1
RELA	RELA	Hs01042010_m1
RPL11	RPL11	Hs00831112_s1
SP1	SP1	Hs00916521_m1
STAT3	STAT3	Hs01047580_m1
STAT5B	STAT5B	Hs00560035_m1
STK11	LKB1	Hs00975988_m1
TBP	TBP	Hs00427620_m1
TCF7L2	TCF7L2	Hs01009044_m1
TFDP1	DP1	Hs00955491_gH
TFDP2	DP2	Hs00963605_m1
TP53	P53	Hs01034249_m1
TRIM28	TIF1B	Hs00232212_m1
YY1	YY1	Hs00231533_m1
ZBTB17	MIZ1	Hs01114794_g1

S6 Table. Literature review leading to 303 initial genes of interest. PID and FunCoup refer to [37] and [39], respectively, updated and online at https://www.ndexbio.org/ and http://funcoup.sbc.su.se/.

Gene/protein	Source
sae1	Amente 2012
usp2	Benassi 2012
ybx1	Bommert 2012
cd19	Chung 2012
srsf1	Das 2012
brd4, brdt, brd2, brd3	Delmore 2011
gata2, gtf2f1, rad21, smc3, ctcf, zbtb7a	ENCODE
cdk9, top2a, topbp1, ddx20, fbl, hnrnpf, eif3e, lmna, nono, psma1, nup153, carm1, mybbp1, cct5, nol11, calm1, calm3, brd8, mms19, mapk1, ep400, usp28, actb, eml3,gcn1l1	Funcoup
cebpb	Funcoup,
gnl3	Funcoup, Zwolinska 2012
slc7a	Hayashi 2012
uba2	Kessler 2012
pfdn5	Kimura 2007
gpc3	Li 2012
hif1a	Doe 2012
pirh2	Hakem 2011

sirt1	Menssen 2012
ezh2, eed, suz12	Neri 2012
yeats4	Piccinni 2011
bag2	Zhang 2012
mxd1, mxi1, mcd3	Luscher 2012
braf, kras	Magudia 2012
nampt, kiaa1967	Menssen 2012
L-myc, N-myc, mycnos	Myc family
ACP5, APEX1, CCKBR, CDC25C, CKS2, ACBP, DKC1, FASN, H2AFZ, HSPA8, HSPE1, MGST1, RPL3, RPS16, SRM, SURF6, JTV1, SFRS2, RPL5	myccancergene.org
rnf115	Narita 2012
stub1	Paul 2013
myc, max, eno1, eno1, myb, bysl, miz-1, miz-1, cebpa, cebpa, cbp, cbp, akt1, e2f4, p107, p53, aes, ikk alpha, ctbp, sp1, sp1, tbp, tcf1, tle1, yy1, mycbp, beta catenin, beta catenin, e2f3, foxm1c, jun, jun, rpl11, dp2, dvl3, p300, p300, er alpha, er alpha, gcn5, gcn5, gcn5, irf-8, jund, nfkappab1, nicd, rb1, rela, ski, brm, stat5a, stat5b, lkb1, dp1, aib1, ncor1, tif1b, cyclin b1, creb1, brca1, id2, id2, ubf, wnt5a, survivin, cad, cyclin d2, cebpd, hsp70, hsp60, trrap, trrap, trrap, bax, bcat1, bcl2, bmi1, bn51, cdc25a, cdk4, p21cip1, rcc1, eif2a, eif4a1, eif4e, eif4g1, hmgcs2, hsp90, irp2, kir3dl1, ldha, mmp9, pro-mmp9, nbs1, nucleolin, nme1, odc, pfkm, serpini1, ptma, rantes, shmt1, glut1, snai1, taf4b, tert, tfr, tk1, fra1, ddx18, mta1, aldh9a1, zfp36l1, p27kip1, clusterin, col1a2, chop, dntt, erbb2, fth1, gtf2h2, alpha6 integrin, beta1 integrin, beta4 integrin, galectin-1, pdgfrb, ppp2r4, psoriasin, sfrp1, tsc2, csde1, smad4, smad4, smad2, smad3, smad3, hmgiy, noxa, p15ink4b, hdac3, gfi1, pim1, baf53, baf53, taf10, taf10, taf12, taf12, spt3, spt3, tip49a, tip49a, staf65g, staf65g, nfya, nfyb, nfyc, p14arf, pml4, zo2, hect9, skp2, gsk3beta, pin1, ppp2ca, pp2a b56alpha, axin1, dleu2, gadd45a, pak2, nramp1, spi1, hdac1, cyclin d1, c-flip, dnmt3a, taf9, taf9, brg1, C-flip p12, C-flip p43, cbp/p300, dyskerin, e2, E2f1-3, fbw7-alpha/usp28, smad2-3-active, tert, gapdh, dleu1, ndrg1, tip60, tip60, mad4, tip49b, tip49b, prdx3, pdcd10, dkk1, tnik, peg10, tmeff2, sin3a, hbp1, fbw7-gamma, ndrg2, gpat, myct1, sfxn3, tcf3, jpo1, tmem126a, mina53, mimitin, aeg-1, lin28b, mirlet7g, mir141, mir146a, mir17, mir200a, mir22, mir23b, mir26a1, mir26a2, mir26b, mir34a, mir9-3, mir338, mir429, nme2	PID
npm1	PID, Funcoup
foxo3a	PID, Peck 2013
stat3	PID; ENCODE indicated this bound MYC
tcf4	PID; Sur 2012
smarca4	Romero 2012; also, ENCODE indicated that it bound MYC
cdk1, cdk2	Goga 2007, Campaner 2010, García-Gutiérrez 2019
oct4, sox2, klf4	Qi 2007
tp63	Wu 2012
dlc1	Zimonjic 2012
